# Behavioral “bycatch” from camera trap surveys yields insights on prey responses to human‐mediated predation risk

**DOI:** 10.1002/ece3.9108

**Published:** 2022-07-17

**Authors:** A. Cole Burton, Christopher Beirne, Catherine Sun, Alys Granados, Michael Procko, Cheng Chen, Mitchell Fennell, Alexia Constantinou, Chris Colton, Katie Tjaden‐McClement, Jason T. Fisher, Joanna Burgar

**Affiliations:** ^1^ Wildlife Coexistence Lab, Department of Forest Resources Management University of British Columbia Vancouver British Columbia Canada; ^2^ School of Environmental Studies University of Victoria Victoria British Columbia Canada

**Keywords:** animal behavior, caribou conservation, habitat disturbance, landscape of fear, predator–prey, remote camera, ungulate ecology, wildlife management

## Abstract

Human disturbance directly affects animal populations and communities, but indirect effects of disturbance on species behaviors are less well understood. For instance, disturbance may alter predator activity and cause knock‐on effects to predator‐sensitive foraging in prey. Camera traps provide an emerging opportunity to investigate such disturbance‐mediated impacts to animal behaviors across multiple scales. We used camera trap data to test predictions about predator‐sensitive behavior in three ungulate species (caribou *Rangifer tarandus*; white‐tailed deer, *Odocoileus virginianus*; moose, *Alces alces*) across two western boreal forest landscapes varying in disturbance. We quantified behavior as the number of camera trap photos per detection event and tested its relationship to inferred human‐mediated predation risk between a landscape with greater industrial disturbance and predator activity and a “control” landscape with lower human and predator activity. We also assessed the finer‐scale influence on behavior of variation in predation risk (relative to habitat variation) across camera sites within the more disturbed landscape. We predicted that animals in areas with greater predation risk (e.g., more wolf activity, less cover) would travel faster past cameras and generate fewer photos per detection event, while animals in areas with less predation risk would linger (rest, forage, investigate), generating more photos per event. Our predictions were supported at the landscape‐level, as caribou and moose had more photos per event in the control landscape where disturbance‐mediated predation risk was lower. At a finer‐scale within the disturbed landscape, no prey species showed a significant behavioral response to wolf activity, but the number of photos per event decreased for white‐tailed deer with increasing line of sight (m) along seismic lines (i.e., decreasing visual cover), consistent with a predator‐sensitive response. The presence of juveniles was associated with shorter behavioral events for caribou and moose, suggesting greater predator sensitivity for females with calves. Only moose demonstrated a positive behavioral association (i.e., longer events) with vegetation productivity (16‐day NDVI), suggesting that for other species bottom‐up influences of forage availability were generally weaker than top‐down influences from predation risk. Behavioral insights can be gleaned from camera trap surveys and provide complementary information about animal responses to predation risk, and thus about the indirect impacts of human disturbances on predator–prey interactions.

## Graphical abstract

Camera traps provide an emerging opportunity to investigate variation in animal behaviors across gradients of disturbance. We used camera trap data to show that caribou, moose, and white‐tailed deer exhibited behavioural patterns consistent with predator‐sensitive foraging induced by anthropogenic habitat disturbance in northern boreal forests.

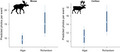

## 1 INTRODUCTION

Wild animals face growing pressure from human land uses and activities, such that few species or ecosystems are untouched by human influence (Díaz et al., 2019; Venter et al., 2016). As landscapes become increasingly human‐dominated, wildlife managers work to balance risks to wildlife with (often) competing human interests, and so seek the best available information on wildlife responses to human disturbances. Impacts to wildlife are often measured as direct effects on animal abundance or distribution, and human‐induced changes in such population‐ and community‐level patterns are widespread (e.g., Ceballos et al., 2017; Newbold et al., 2015). However, not all human impacts are direct. There is increasing evidence of the importance of indirect effects mediated through changes in animal behavior (Ciuti et al., 2012; Suraci et al., 2019), including spatial patterns of habitat use, movement, and foraging (Berger‐Tal et al., 2011; Gaynor et al., 2021). For example, species may shift their diel activity pattern to avoid interaction with humans, with potential consequences for resource acquisition (Gaynor et al., 2018; Shamoon et al., 2018). Ultimately, human‐induced changes in key behaviors, such as movement, can affect ecosystem services (Tucker et al., 2021) and serve as an early warning of impending demographic effects (Middleton et al., 2013). Understanding the indirect impacts of human disturbances on animal behavior is critical for developing a fuller picture of wildlife responses to anthropogenic change, and ultimately for developing effective management actions.

Human impacts to animal behavior are likely to have knock‐on effects on interactions among species within communities. In particular, predator and prey species may respond differently to human disturbances, leading to altered predator–prey dynamics in human‐impacted ecosystems (Muhly et al., 2011; Smith et al., 2015). Such altered dynamics may be particularly significant for the conservation of endangered prey species, such as woodland caribou (*Rangifer tarandus caribou*). Across North America, anthropogenic habitat changes have altered predator–prey dynamics, leading to increases in prey species such as moose (*Alces alces*) and white‐tailed deer (*Odocoileus virginianus*), which in turn drive increases in their main predator, gray wolf (*Canis lupus*), and corresponding declines in threatened caribou populations (a process known as apparent competition; Festa‐Bianchet et al., 2011; Latham et al., 2011; Wittmer et al., 2005). Declines in caribou have been further exacerbated by increased predation efficiency associated with industrial disturbances such as roads and seismic lines, which facilitate movement by wolves and potentially other predators (DeMars & Boutin, 2018; Dickie et al., 2017).

The direct and indirect effects of predation are key drivers of dynamics for caribou and their interacting species, as they are in many other conservation contexts (Gaynor et al., 2021; Serrouya et al., 2015). Management efforts to recover caribou have focused primarily on reducing predation risk by reducing predator abundance (Serrouya et al., 2019) and restoring habitat to reduce predator movements (Beirne et al., 2021; Tattersall et al., 2020). If successful, such efforts will alter the “landscape of fear” for prey species, leading to changes in risk‐related prey behaviors (Laundré et al., 2010). For example, prey are expected to avoid or move more quickly through habitats with higher predation risk, and exhibit more secure behaviors in less risky habitat (e.g., resting, foraging; Dickie et al., 2020). Nevertheless, quantifying such behavioral changes in response to management actions has been difficult, given the need to collect concurrent data on multiple interacting species. In the caribou context, Dickie et al. (2020) found evidence that prey species moved more quickly on linear features (e.g., seismic lines), where predation risk was assumed to be higher, and more slowly in areas with lower predation risk and greater forage availability. Yet such multispecies telemetry‐based studies are costly and invasive, and may not be easily linked to management actions if, for example, collared individuals do not interact with restored habitat features, or relocation intervals are too coarse to support strong behavioral inferences (Buderman et al., 2021). Similarly, direct behavioral observation of wildlife (e.g., Bøving & Post, 1997) is often expensive or impractical, particularly in remote, forested environments such as the habitats of woodland caribou.

Camera trap (CT) surveys provide a promising means of monitoring behavioral responses of terrestrial mammals to management actions. Camera traps are increasingly used to study wildlife as they provide a cost‐effective and noninvasive method of surveying multiple, interacting species (Steenweg et al., 2017). To date, CT studies have typically focused on quantifying species distributions, population densities, and habitat use (Burton et al., 2015), but more recently their value for generating inferences on behavior has been highlighted (Caravaggi et al., 2017; Smith et al., 2020). For example, CTs have been used to quantify behaviors such as foraging time and social interactions (Cherry et al., 2015; Stone et al., 2017), diel activity and temporal avoidance (Frey et al., 2020; Higdon et al., 2019), risk tolerance (Stewart et al., 2016), curiosity (Kalan et al., 2019), and vigilance (Altendorf et al., 2001; Le Saout et al., 2015; Schuttler et al., 2017). Nevertheless, such examples remain the exception; most CT studies do not infer behaviors, and when they do, there is little guidance on deriving reliable measures of behavior from collected photos.

Here, we use CT data to explore the responses of ungulate prey to perceived risk in the context of caribou conservation in the boreal forests of northeastern Alberta. We tested whether caribou and their apparent competitors (moose, white‐tailed deer) were responding to a landscape of fear by altering behaviors in response to variation in human‐mediated predation risk. Specifically, we tested whether camera trap‐derived measures of “secure” or “risk‐averse” behaviors were consistent with predictions of predator‐sensitive foraging. We assumed that prey species would travel faster and linger less in areas of higher perceived predation risk (risk‐averse), and conversely travel more slowly and linger more in areas of lower risk (secure). First, we compared prey behaviors across two landscapes that varied in habitat disturbance and relative abundance of the dominant predator (gray wolf), and thus in assumed predation risk. Second, we tested whether spatial variation in prey behaviors within the riskier landscape was better explained by the top‐down influence of predation risk or the bottom‐up influence of habitat (forage availability). At both scales, differences in predation risk were inferred based on variation in the relative abundance of wolves and in the prevalence or characteristics of anthropogenic disturbance features known to influence wolf movement.

## 2 METHODS

### 2.1 Study area

**FIGURE 1:**
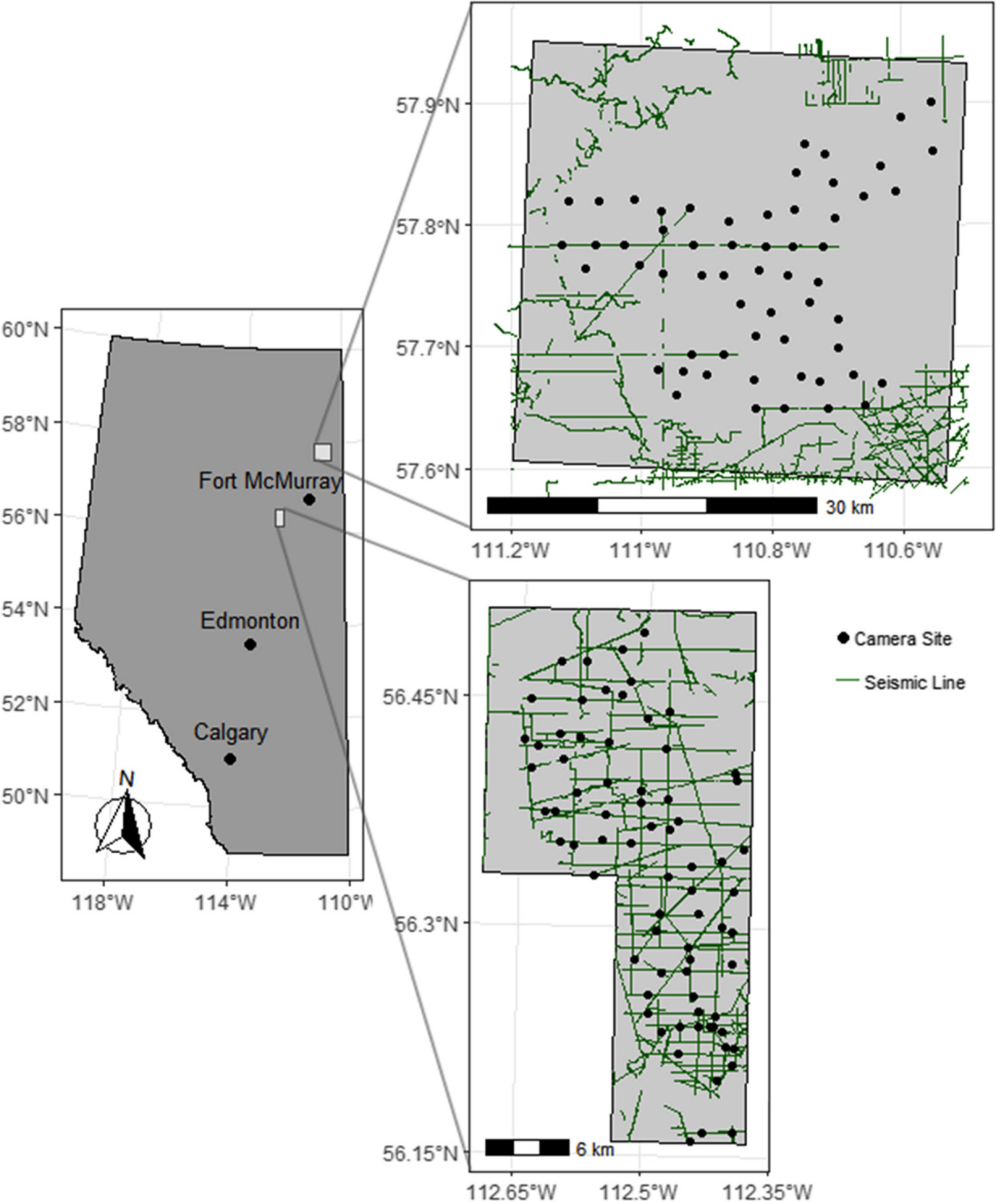
Location of the Algar and Richardson study areas in northeastern Alberta, Canada. Green line segments indicate seismic lines. Filled circles show the 73 camera trap sites in Algar that operated from November 2015 to November 2019 and the 57 camera trap sites in Richardson that operated from November 2017 to November 2019

Woodland caribou inhabit forested environments that are increasingly impacted by industrial and natural disturbances (Festa‐Bianchet et al., 2011; Johnson et al., 2020). In particular, many of Alberta's northern boreal forests have been extensively modified from disturbances driven by oil and gas extraction and forest harvest (Fisher & Burton, 2018; Pickell et al., 2015). Our CT surveys were conducted in two northeastern Alberta landscapes with broadly similar vegetation characteristics but varying in anthropogenic disturbance: the intermediately disturbed Algar caribou sub‐range (part of the East Side Athabasca River range) and the less disturbed Richardson caribou range (Figure 1; Hervieux et al., 2013).

The Algar sampling area spans ca. 570 km^2^ and is located 70 km southwest of Fort MacMurray, Alberta (Figure 1). It is part of the western sedimentary basin of the Canadian boreal forest, containing lowlands with mature black spruce (*Picea mariana*) and tamarack (*Larix laricina*) wetlands, and white spruce (*Picea glauca*), aspen (*Populus tremuloides*) and jack pine (*Pinus banksiana*) uplands. Seismic lines and other features related to oil and gas extraction (e.g., pipelines, well pads) are the dominant anthropogenic disturbance in the Algar landscape, which has an intermediate density of seismic lines (ca. 2.0 km/km^2^) relative to other areas in Alberta's oil sands region. The area was subject to linear restoration treatments between 2012 and 2015, through a combination of planting black spruce seedlings, adding coarse woody debris, and mechanical mounding of the soil to promote vegetation growth and discourage predator use (Tattersall et al., 2020).

The Richardson study area is approximately 200 km northeast of the Algar study area (Figure 1) and covers ca. 1500 km^2^. The landscape has substantially less anthropogenic disturbance than the Algar landscape (e.g., seismic line density of ca. 0.4 km/km^2^) and is dominated by a mosaic of burned and unburned patches of upland jack pine and black spruce bog, which are regenerating after a large fire in 2011 (Burgar & Burton, 2019).

### 2.2 Camera trap surveys

We deployed a single CT (Reconyx HyperFire PC900, Holman, WI) at each sampling site across the Algar and Richardson study areas in stratified random designs. The primary objectives of the surveys were to assess the distribution and relative abundance of medium‐ and large‐bodied mammals in relation to landscape characteristics, particularly industrial disturbances like seismic lines. In the Algar study area, 73 CTs were deployed between November 2015 and November 2017, with year‐round sampling continuing until November 2019. Sixty CT sites were on seismic lines and 13 were off of seismic lines. On‐line sites were further stratified by restoration category (treated, regenerating, unrestored control, human use; details in Beirne et al., 2021). In the Richardson study area, CTs were deployed in November 2017 and 2018 at 57 sites stratified by in (27) vs. out (30) of burned area and on (18) vs. off (39) of a seismic line, with year‐round sampling continuing until November 2019 (Burgar & Burton, 2019). In both study areas, cameras were left in place and sampled continuously once deployed (mean sampling days per station was 1024 in Algar and 508 in Richardson).

At all sampling sites, CTs were placed on a tree 2–5 m from the edge of a seismic line or game trail, facing across the line (i.e., perpendicular to expected direction of animal travel), at a height of approximately 0.8 m above the ground (range = 0.7–1.1 m), targeting medium‐ to large‐bodied mammals without bait or lure. One picture was taken per motion trigger with no delay between subsequent triggers and sensitivity was set to maximum with a fast shutter speed. CTs were active for 24 h per day with no quiet period. One timelapse picture was taken each day at noon to ensure camera function (any camera‐days with snow occluding the lens were excluded, but this rarely occurred). All methods for wildlife monitoring were approved by the Canadian Council of Animal Care administered by the University of British Columbia (protocol A17‐0035).

At each CT site, we quantified line of sight (LOS) as an indicator of habitat openness, under the assumption that ungulates would be less likely to use more open habitats with greater visibility, where the risk of predation may be higher (Dickie et al., 2020). Previous analysis showed that LOS was a useful descriptor of variation in linear feature conditions with respect to wildlife use in the Algar landscape (Beirne et al., 2021). We measured LOS (in m) along the seismic line or game trail perpendicular to the camera. We used a laser rangefinder to take three distances to the first sight barriers to the left and right of each camera, which were then averaged across each visit where readings were taken to give a single LOS value for each camera location. We were unable to measure seasonal variation in LOS, although we did include a satellite‐based measure of seasonal variation in vegetation (described below).

We processed CT images by identifying the focal ungulate and predator species (woodland caribou, white‐tailed deer, moose, wolf). For ungulate species, we counted the number of unique individuals (i.e., group size) and classified images by the sex (male, female) and age class (adult or juvenile, i.e., young of the year) of visible individuals. We characterized observed behavior for ungulate prey species in each image as either Secure (resting; foraging with food visible in its mouth or its head down with open mouth; inspecting camera with the face covering ≥20% the image), or Traveling (animal seen walking past camera; Figure S1 in Appendix S1). Species identifications were made from images within Camelot software (Hendry & Mann, 2018); any uncertain identifications were excluded from analysis. For each species, we defined a detection event at a given site as a sequence of images separated by no more than 15 min between consecutive images (Rovero & Spitale, 2016). This 15‐min threshold was based on identifying a consistent gap time between subsequent images across all focal species (Figure S2 in Appendix S1).

### 2.3 Modeling ungulate behavior

We evaluated three ways of distinguishing “at risk” from “secure” behaviors for each detection event of ungulate prey: classification, event duration, and number of photos per event (Appendix S1). We first considered whether the majority of photos in an event demonstrated Secure or Traveling behaviors (classified following above definitions), but determined that behavioral classes could be difficult to discern over short event durations or when multiple behaviors were exhibited within a single event. We next inferred that risk‐averse behaviors, such as traveling through an area without foraging, would result in detection events of shorter duration with fewer photos, whereas more secure behaviors (e.g., foraging, resting, inspecting camera) would have longer detection events and more photos, as the animal lingered in front of the camera. We found that large time intervals between detections could inflate event durations and skew the observed distribution (resulting in poor model fit; Table S4 and Figure S19 in Appendix S1). Therefore, while the three different response variables were correlated, we focused our analysis on the number of photos per detection event as the primary response of interest. We found it to be less sensitive to subjective decisions during classification, and thus consider it to be a more objective and easier to calculate index of estimated variation in risk‐related behavior (see Appendix S1 for further details).

### 2.4 Landscape‐level comparison

Risk of wolf predation on ungulate prey is influenced by wolf abundance and hunting efficiency (i.e., numerical and functional responses; Serrouya et al., 2015), and the latter is expected to increase with increasing density of linear features in a landscape, since wolves use those features to hunt (e.g., roads, seismic lines; Dickie et al., 2017). The more disturbed Algar landscape was considered to have higher predation risk than the Richardson landscape, given its greater relative abundance of wolves (0.7 wolf detection events per 100 CT days in Algar vs. 0.1 in Richardson; see Results) and higher seismic line density (2.0 km/km^2^ in Algar vs. 0.4 km/km^2^ in Richardson; Figure 1).

Accordingly, we hypothesized that ungulate prey would show more risk‐averse behavior in the higher predation risk Algar landscape, and thus we predicted that the number of photos per detection event of ungulates would be lower in Algar than in Richardson. We tested this prediction for caribou and moose, which had sufficient detections in both landscapes (white‐tailed deer are at the northern limit of their range in the Richardson landscape and were not detected frequently enough to compare behaviors across landscapes). We used negative binomial generalized linear mixed models (GLMMs) to model the number of observations per event for caribou or moose as a function of the landscape (Algar or Richardson as a binary predictor variable, capturing the landscape‐level differences in disturbance‐mediated wolf activity). We included CT site as a random intercept to account for potential correlated behavior due to unmodeled site conditions, and group size per event as an offset to control for the fact that larger groups are likely to have more photos per event (i.e., we wanted to focus on individual behaviors rather than group formation, but not discard the detections of groups). Our specific model took the form:

$$\log\left({\text{photos}}_{\mathrm{jk}}\right)=\beta_{0j}+\beta_{1}{\text{StudyArea}}_{\mathrm{jk}}+\log\left(\text{groupSiz}e_{\mathrm{jk}}\right)+\epsilon$$

for a species (caribou or moose) at site *j* = 1, 2, …, 131 (73 sites in Algar + 57 in Richardson), and detection event *k* = 1, 2, …, *K* (see Results for total detection events for each species).

### 2.5 Site‐level analysis

While prey may show population‐level behavioral differences between landscapes that differ in disturbance‐mediated predation risk, they may also show finer‐scale variation in behavior due to heterogeneity in risk within a landscape. We assessed variation in behavior among detection events as a function of site‐level variation in estimated predation risk and habitat quality within the more disturbed Algar landscape for caribou, moose, and white‐tailed deer. We focused on the Algar survey as it had more detections of wolves and white‐tailed deer, and the linear restoration treatments introduced more site‐level variation in potential predation risk than in the Richardson landscape. We used GLMMs to test the prediction that the number of photos per detection event would decrease with increasing predation risk. To quantify site‐level variation in the latter, we used wolf relative abundance estimated from each CT (wolf detection events per 100 camera‐trap days) measured at both fine (16‐day) and coarse (full sampling period) temporal scales around detection events, which we assumed to reflect short‐term and long‐term variation in predation risk, respectively (e.g., risky times vs. risky places; Dröge et al., 2017). As an additional indicator of potential disturbance‐mediated predation risk, we included the LOS at each site, which was assumed to indicate prey visibility to wolves (i.e., correlate with predator efficiency). LOS was measured as described above and ranged from 23 to 1039 m (mean ± 1 SD = 387 ± 281). We also considered the presence of juveniles in prey detection events as an additional model predictor, because juveniles may be more susceptible to predation than adult prey and therefore cause groups to exhibit more risk‐averse behaviors (Table 1).

**TABLE 1:** Site‐level predictor variables used in generalized linear mixed models to explain variation in behavior of caribou, moose, and white‐tailed deer, as measured by the number of photos per camera trap (CT) detection event, in the Algar landscape. Also given is the temporal scale of each variable (16‐day sampling period or full study period, November 2015–2019) and its hypothesized influence on the behavioral response. With the exception of the “Juveniles” predictor (a binary variable measured as juvenile presence or absence in an event), all variables were standardized to have a mean of 0 and standard deviation of 1

Category	Predictor variable	Description	Temporal scale	Hypothesis
Predator	Line of sight (LOS)	Distance of unobstructed sight along seismic line (m)	Full study period	Greater LOS increases predation risk and reduces photos per event
Predator	Wolf RAI_Full; Wolf RAI_16	Relative abundance of gray wolves (detection events per 100 CT days)	Full study period, 16‐day	Greater relative abundance of wolves increases predation risk and reduces photos per event
Predator	Juveniles	Presence (1) or absence (0) of juveniles in an event	Event	Juveniles experience greater predation risk, so groups with juveniles will have fewer photos per event
Habitat	NDVI_Full; NDVI_16	NDVI value within a 500 m buffer around the site, summed or per 16‐day interval	Full study period, 16‐day	Greater NDVI values indicate more foraging opportunities, which will increase the photos per event
Habitat	Lowland forest	Proportion of lowland habitat within a 500 m buffer around the site	Full study period	Control for broad habitat preferences known to influence prey distribution
Offset	Group size	Observed number of unique individuals per detection event	Event	Control for effect of group size on photos per event

As an alternative hypothesis to the top‐down influence of predation risk on prey behavior, we considered the bottom‐up influence of habitat quality, such that prey behavior may be driven by the need to access forage. We used the Normalized Difference Vegetation Index (NDVI) as proxy for vegetation productivity, which has been shown to be correlated with ungulate foraging (e.g., DeCesare et al., 2012; Merkle et al., 2016). We obtained NDVI from the MOD13Q1 product using the “MODISTools” R package (Tuck et al., 2014), and summarized values within a 500 m buffer around each CT at two temporal scales: (1) the 16‐day interval at which NDVI composites are provided to characterize seasonal vegetation patterns related to wildlife foraging activity (Figure S11 in Appendix S1), and (2) the entire study period (2015–2019) by summing NDVI values to characterize longer term site‐level habitat quality. Over the entire study period, total NDVI in the Algar study area ranged from 34,234 to 549,758 (mean ± 1 SD = 44,191 ± 44,202) while 16‐day NDVI ranged from 0 to 9238 (mean ± 1 SD = 5793 ± 2209) across the 16‐day periods with ungulate detection events. To account for broader differences in habitat type, we also quantified the percentage of lowland habitat within the 500 m buffer around each CT, based on moisture regime and forest type (Tattersall et al., 2020; Table 1).

Model predictor variables were tested for collinearity (none were highly correlated, |*r*| < 0.5; Figure S12 in Appendix S1) and standardized to have a mean of 0 and standard deviation of 1 (with the exception of “Juveniles,” a binary variable measured as juvenile presence or absence in an event). As for the landscape‐level models, we modeled the number of photos per event as a negative binomial response (to account for overdispersion in models run with Poisson distribution) and included site as a random intercept and group size per detection event as an offset. We constructed a full model with all covariates for each ungulate species following the form:

$$\log\left({\text{photos}}_{\mathrm{jk}}\right)=\beta_{0j}+\beta_{1}{\mathrm{LOS}}_{j}+\beta_{2}{{\text{WolfRAI}}_{\text{Full}}}_{j}+\beta_{3}{{\text{WolfRAI}}_{16}}_{\mathrm{jk}}+\beta_{4}{\text{Juveniles}}_{\mathrm{jk}}+\beta_{5}\text{NDVI}\_{\text{Full}}_{j}+\beta_{6}\text{NDVI}\_16_{\mathrm{jk}}+\beta_{7}{\text{Lowland}}_{j}+\log\left(\text{groupSiz}e_{\mathrm{jk}}\right)+\epsilon$$

for a species at site *j* = 1, 2, …, 73, and detection event *k* = 1, 2, …, *K* (see Table 2 for total detection events for each species).

**TABLE 2:** Summary statistics for ungulate detection events from which behavior was inferred, collected from 73 camera traps in the Algar study area between November 2015 and 2019 and 57 camera traps in the Richardson study area between November 2017 and 2019. A detection event was defined as one or more photos of a species at a site with no more than 15 min between consecutive photos. For photos per event and group size, the maximum value is reported (minimum was 1 for all species)

Study area	Species	Sites (%)	Detection events	Photos per event, Mean ± 1 SD (max)	Group size, Mean ± 1 SD (max)	Prop. Events with juveniles
Algar	Deer	56 (0.77)	1370	9 ± 12 (175)	1.2 ± 0.5 (5)	0.06
Algar	Moose	57 (0.78)	418	12 ± 21 (215)	1.3 ± 0.5 (4)	0.16
Algar	Caribou	42 (0.58)	349	10 ± 12 (72)	1.4 ± 1.0 (10)	0.09
Richardson	Deer	2 (0.04)	2	8 ± 6 (12)	1 ± 0 (1)	0
Richardson	Moose	24 (0.42)	110	19 ± 28 (129)	1.2 ± 0.4 (3)	0.17
Richardson	Caribou	28 (0.49)	231	18 ± 33 (292)	2.0 ± 1.5 (9)	0.24

### 2.6 Model implementation

We ran each model in a Bayesian framework with the *brms* (Bürkner, 2017) package in Program R (R Core Team, 2020). Models with default noninformative priors ran for 25,000 iterations for each of 3 chains with a thin rate of 5, after an initial warm up of 2500 iterations. Convergence was confirmed through visual inspection of trace plots and the Gelman–Rubin statistic (Rhat < 1.1; Gelman & Hill, 2006). We assessed model fit using posterior predictive checks with the *pp_check* function and by calculating Bayesian *R* ^2^ (Gelman et al., 2019) using the *bayes_R2.brmsfit* function (Tables S3, S4 and Figures S16–S19 in Appendix S1). We considered there to be strong evidence of an effect when the 95% credible interval (CI) from the posterior distribution did not overlap 0.

## 3 RESULTS

### 3.1 Landscape‐level comparison

A total survey effort of 103,788 CT days (74,364 in Algar and 29,424 in Richardson) yielded more than 100 detection events of each prey species in each landscape, with the exception of white‐tailed deer in Richardson (Table 2). Ungulate species varied in the mean number of photos per detection event, and caribou had the largest average group sizes (Table 2). Wolves were detected at more sites and more frequently in Algar than in Richardson. In Algar, wolves were detected at 75% of survey sites (55 of 73) and had a mean of 0.7 (SD = 1.2) detections per 100 CT days (range = 0.0–5.8). By contrast, in Richardson, wolves were detected at 30% of survey sites (17 of 57), with a mean of 0.1 (SD = 0.2) detections per 100 CT days (range = 0.0–0.8). All focal species were detected at CTs set on and off of seismic lines (Table S1 in Appendix S1)

**FIGURE 2:**
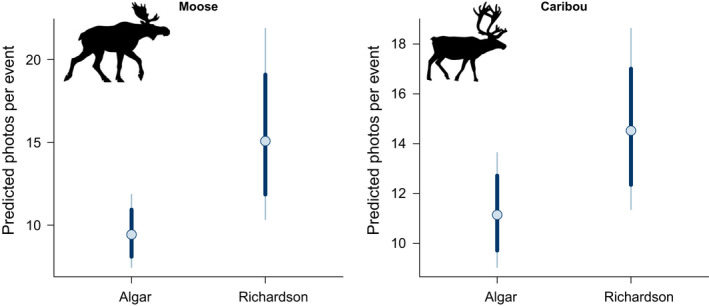
Model‐predicted photos per detection event for moose (left) and caribou (right) from generalized linear mixed models comparing observed detections per event between a landscape with higher disturbance‐mediated predation risk (Algar) and one with less disturbance and lower predation risk (Richardson). Points represent the mean model‐predicted detections per event for each landscape, thick lines denote the 80% credible intervals, and thin lines denote the 95% credible intervals for the predictions

We found strong support for the predicted effect of landscape on the number of moose detections per event (mean effect = 0.47; 95% CI = 0.03–0.92), with 60% more moose detections per event in the less disturbed, lower predation risk Richardson landscape than the more disturbed, higher predation risk Algar landscape (Figure 2). A similar but weaker trend was observed for caribou, with 30% more detections per event in Richardson than Algar (mean effect = 0.26; 95% CI = −0.05 to 0.59; Figure 2)

### 3.2 Site‐level analysis

**FIGURE 3:**
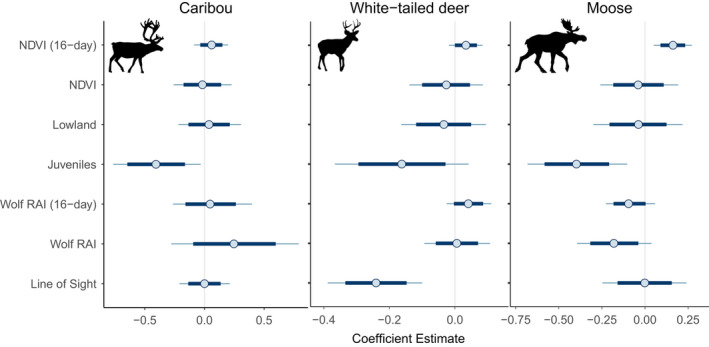
Posterior coefficient estimates for generalized linear mixed models of number of photos per detection event for caribou, white‐tailed deer, and moose as a function of site‐level predation risk (estimated by wolf relative abundance, RAI, at 16‐day and full survey temporal scales, line of sight, and the presence of juveniles) and habitat quality (estimated by NDVI at 16‐day and full survey temporal scales, and percent lowland forest in a 500 m radius around the camera location) within the more disturbed Algar landscape. Points represent the mean coefficient estimates, thick lines denote the 80% credible intervals, and thin lines denote the 95% credible intervals. All predictor variables were standardized to have a mean of 0 and standard deviation of 1 (with the exception of binary presence/absence of juveniles)

We found some support for the predicted influence of estimated predation risk on behavioral responses of ungulate prey within the Algar landscape (Figure 3). LOS was negatively associated with the number of photos per detection event for white‐tailed deer (mean effect = −0.24; 95% CI = −0.39 to −0.10; Figure S13 in Appendix S1), while the presence of juveniles was associated with a significant decrease in the number of photos per detection event for caribou (mean effect = −0.41; 95% CI = −0.77 to −0.03) and moose (mean effect = −0.40; 95% CI = −0.68 to −0.10). Contrary to expectations, the relative abundance of wolves at a site was not a strong or consistent predictor of variation in photos per detection event across ungulate prey species; there was weak evidence of a negative association for moose, but also of a positive association for deer (Figure 3). There was little support for an important role of habitat on behavioral responses, with only moose showing strong evidence for the predicted increase in photos per event with increasing NDVI, at the 16‐day temporal scale (Figure 3; Figure S13 in Appendix S1).

## 4 DISCUSSION

Our study highlights the ability of CT surveys to collect data on variation in animal behavior across space and time, even in studies designed for other purposes, such as estimating habitat use or population abundance. We used the number of photos per detection event—a simple statistic easily derived from CT surveys—to quantify behavioral variation for three large ungulate prey species, and tested the hypothesis that prey behavior would be more risk‐averse (i.e., prey moving more quickly with less time foraging or resting) in areas with higher predation risk. We found evidence consistent with behavioral responses to predation risk for caribou, moose, and white‐tailed deer across variation in wolf relative abundance and anthropogenic disturbance at two spatial scales in boreal forests of northeastern Alberta, although responses varied across species. Our findings yield insights into predator–prey interactions relevant to the management of threatened caribou, and advance the application of camera trapping to the study of wildlife behavior.

At the population scale, caribou and moose were frequently detected in two landscapes that varied in wolf relative abundance and seismic line density. As predicted, both species had fewer detections per event in the higher risk Algar landscape relative to the lower risk Richardson landscape. These results were consistent with the hypothesis that prey would move more quickly and forage less in a landscape where their primary predator was relatively more abundant and could move more efficiently (Dickie et al., 2020; Kie, 1999; Laundré et al., 2010). That white‐tailed deer were rarely detected in the low wolf activity Richardson landscape is also consistent with the apparent competition hypothesis, which posits that increases in deer are driving increased wolf predation on caribou in northeastern Alberta (Dawe et al., 2014; Latham et al., 2011). That is, in such areas where deer have only marginally expanded their northern distribution and density, wolf populations have not increased and predation risk on caribou is presumably lower. If changes in climate and land use continue to drive deer expansion farther north, the Richardson caribou population (and other northern populations) may face increasing risk from predation as wolves follow deer. In such rapidly changing and remote northern forests, CT monitoring can provide a valuable means of early detection of shifts in wildlife behaviors that may foreshadow shifts in species interactions, abundances, and ultimately ecosystem functions (Barrueto et al., 2014; Juanes, 2018; Shamoon et al., 2018).

At the finer scale of habitat use within the disturbed Algar landscape, we predicted that higher relative abundance of wolves and greater visibility along seismic lines, along with the presence of juvenile prey, could all increase prey vulnerability to predation and elicit risk‐averse responses at the scale of individual CT sites. We found that the presence of juveniles in prey groups was the only predictor associated with fewer photos per event across all three species (Figure 3), suggesting that females accompanied by more vulnerable young may travel faster or forage less on seismic lines to reduce perceived risks. This response was estimated with less certainty for white‐tailed deer, but, unlike moose and caribou, deer showed a strong negative behavioral response to increasing visibility (LOS). We suggest that this stronger aversion to presumably riskier line conditions could be because deer are generally less well adapted to predator avoidance within the wetland terrain characteristic of these northern boreal forests. As deer have more recently expanded into this region from southern forests (Dawe et al., 2014; Fisher et al., 2020), they may be more reliant on movement along the cleared linear disturbances and accordingly be more risk averse in areas of higher visibility. In contrast, caribou are known to spatially separate from wolves and their apparent competitors to reduce predation risk (James et al., 2004), which may have mitigated the need for more risk‐averse behavior along seismic lines. Indeed, posthoc analysis suggested that caribou were spatially segregating, as the relative abundance of wolves was lower where caribou were detected compared to where deer and moose were detected. We observed this over both the full study period (mean and maximum wolf detections per 100 CT days were 0.38 and 3.2, respectively, where caribou were detected vs. 0.88 and 5.8 where deer and/or moose were detected) and the 16‐day interval (mean and maximum of 0.004 and 0.27 where caribou were detected vs. 0.009 and 0.33 where deer and/or moose were detected). Our focus on prey behavior in riskier areas did not explicitly account for avoidance of those areas altogether; we recommend that future research seek to integrate CT measures of habitat use (e.g., Tattersall et al., 2020) with measures of behavior given use, such as those we present here. Additionally, we suggest that other useful directions for research include evaluating rates of predation on ungulate species across a gradient of distances from linear disturbances, and further evaluating differences in predator‐avoidance behaviors between native prey species and those that have recently expanded their distributions (Le Saout et al., 2015; Twining et al., 2020).

Contrary to our expectations, none of the prey species showed a strong behavioral response to the observed variation in relative abundance of wolves within Algar. It is possible that the large ranges of movement by wolves, which can extend beyond the size of the Algar study area (Dickie et al., 2022), render CT detection rates a poorer indicator of relative predation risk at the local site scale than they are at the landscape scale. This highlights a key challenge with inferring species interactions from correlational data on species co‐occurrences (Blanchet et al., 2020), as underlying mechanisms of interaction are difficult to distinguish by mutually exclusive hypotheses. Testing patterns for consistency with mechanistic hypotheses is a first step, but triangulating results across different ways of estimating interactions is important, and experimental approaches should be pursued where possible (Hik, 1995; Smith et al., 2020).

As with the inferred behavioral responses to our estimates of indirect predation risk, prey responses to measures of habitat quality were mixed. Variable responses across species may underscore differences in their foraging strategies as well as their need to balance trade‐offs between forage acquisition and predator avoidance (Berg et al., 2021; Kie, 1999; Martin & Owen‐Smith, 2016). Ungulate foraging is expected to track spatial and temporal variation in forage availability (Merkle et al., 2016), so we assumed that foraging behavior would lead to more photos per detection event in habitats with greater forage, which we estimated using NDVI. However, satellite‐derived NDVI may not have accurately captured the patterns of understory vegetation that caribou and deer depend on more so than moose (Sun et al., 2021). Indeed, only moose showed the expected positive association with 16‐day NDVI as an index of seasonal forage availability (Figure 3). Still, none of the species showed evidence of a behavioral response to the broad habitat type (upland vs. lowland) or overall productivity (NDVI for the full sampling period), which suggests that perceived predation risk may have a stronger influence than forage availability on prey behavior at this scale. Understanding trade‐offs between attraction to forage and avoidance of predators, and the spatial scales at which they occur, is fundamental to predicting species responses to caribou management actions, such as habitat restoration and predator control. Further investigation of direct measures of forage availability, resource selection, and mortality from predation is warranted for ungulates in this system (Darlington et al., 2022; Finnegan et al., 2018; McKay et al., 2021).

Ultimately, animal behaviors are complex and influenced by many interrelated factors (Creel et al., 2014; Moll et al., 2016). Indirect measures of behavior derived from CT “bycatch” data provide only an imperfect glimpse into this complexity, yet they remain a rich source of information to be explored. Sampling coverage by CT surveys has grown rapidly (Chen et al., 2022), and our simple index of behavior was easy to calculate from data collected to estimate species distributions and abundances. We note that our results were not always consistent across the three indicators we explored (Figures S14 and S15 in Appendix S1) and we encourage further inquiry into the strengths and weaknesses of different ways to measure behavior from CT data—for example, using video recordings to estimate movement speed (Rowcliffe et al., 2016) and machine learning to automate behavioral classifications (Palencia et al., 2021). We recommend more evaluation of the role of group size in affecting animal behavior (e.g., shared vigilance; Laundré et al., 2001; Olson et al., 2015), particularly for species that exhibit herding behaviors like caribou, as group sizes were low in our study and thus assumed to have limited effect. We also emphasize the potential importance of animal responses to cameras, which can influence interpretations of natural behaviors (Caravaggi et al., 2020; Meek et al., 2016) and were not directly considered in this study. We assumed animal curiosity with respect to cameras would reflect secure behavior, and apprehension toward cameras would reflect risk‐averse behavior, and thus both would be consistent with our interpretation of photos per event. Nevertheless, future studies could further probe these assumptions relative to alternatives, such as neophobic responses leading to camera avoidance.

Camera traps have rapidly become a primary survey tool in wildlife research and management, with their sampling coverage expanding around the world (Chen et al., 2022). With the emergence of CT networks at regional (e.g., WildCAM, Granados et al., n.d. in review) and global (e.g., Wildlife Insights, Ahumada et al., 2020) scales, there is increasing coordination and synthesis of camera detection data across large spatial and temporal scales. While many CT surveys focus inferences primarily on occupancy or abundance (Burton et al., 2015), we highlight that these same surveys can also generate data on variation in animal behaviors across diverse environmental contexts. We suggest that such behavioral data can provide early indicators of population and community responses to environmental changes, yielding warnings of impending changes in vital rates and species interactions, and enriching our understanding of cumulative environmental effects in complex ecological systems (Burton & Chetkiewicz, 2021; Greggor et al., 2016). Early warnings and improved understanding are urgently needed in efforts to protect and recover threatened species, such as woodland caribou in Canada, as their declines continue despite considerable conservation investment (Hebblewhite, 2017; Superbie et al., 2022). Our approach opens the door to further evaluation of behavioral responses to anthropogenic disturbances and management actions across large scales, and better integration of indicators of behavior with other camera trap measures such as activity, co‐occurrence, and abundance (Burgar et al., 2019; Frey et al., 2022; Naidoo & Burton, 2020).

## AUTHOR CONTRIBUTIONS

**A. Cole Burton:** Conceptualization (equal); data curation (equal); funding acquisition (lead); investigation (lead); methodology (equal); project administration (lead); resources (lead); supervision (lead); writing – original draft (equal); writing – review and editing (lead). **Christopher Beirne:** Conceptualization (equal); data curation (equal); formal analysis (equal); methodology (equal); writing – original draft (equal); writing – review and editing (supporting). **Catherine Sun:** Conceptualization (equal); data curation (equal); formal analysis (equal); methodology (equal); writing – original draft (equal); writing – review and editing (supporting). **Alys Granados:** Conceptualization (supporting); data curation (supporting); formal analysis (equal); methodology (equal); writing – original draft (equal); writing – review and editing (supporting). **Michael Procko:** Conceptualization (supporting); formal analysis (supporting); methodology (supporting); writing – original draft (supporting); writing – review and editing (supporting). **Cheng Chen:** Conceptualization (supporting); data curation (supporting); formal analysis (supporting); methodology (supporting); writing – original draft (supporting); writing – review and editing (supporting). **Mitchell Fennell:** Conceptualization (supporting); formal analysis (supporting); methodology (supporting); writing – original draft (supporting); writing – review and editing (supporting). **Alexia Constantinou:** Conceptualization (supporting); formal analysis (supporting); methodology (supporting); writing – original draft (supporting). **Chris Colton:** Conceptualization (supporting); methodology (supporting); writing – original draft (supporting). **Katie Tjaden‐McClement:** Conceptualization (supporting); methodology (supporting); writing – original draft (supporting); writing – review and editing (supporting). **Jason T. Fisher:** Funding acquisition (supporting); project administration (supporting); resources (supporting); writing – review and editing (supporting). **Joanna Burgar:** Data curation (supporting); funding acquisition (supporting); project administration (supporting); writing – review and editing (supporting).

## CONFLICT OF INTEREST

The authors have no conflicts of interest to declare.

## Supporting information


Appendix S1

## Data Availability

Data and R code are available on Dryad at https://doi.org/10.5061/dryad.98sf7m0mg.
