# Supplementary material for: Behavioral “bycatch” from camera trap surveys yields insights on prey responses to human‐mediated predation risk
Source: Ecol Evol. 2022 Jul 17;12(7):e9108. doi: 10.1002/ece3.9108 (PMC9288887; doi:10.1002/ece3.9108)
Supplement: Supplementary file 1 — Appendix S1 [file ECE3-12-e9108-s001.docx]

**APPENDIX**

**SUPPLEMENTARY METHODS**

We considered three ways of quantifying behavioral variation at camera trap (CT) locations: a) classifying observed behaviors as either “at risk” (i.e., travelling) or “secure” (i.e. lingering), b) calculating the temporal duration of detection events, i.e. sequences of consecutive images of the same individual(s), and c) calculating the number of photos per event (Fig. S1).

**
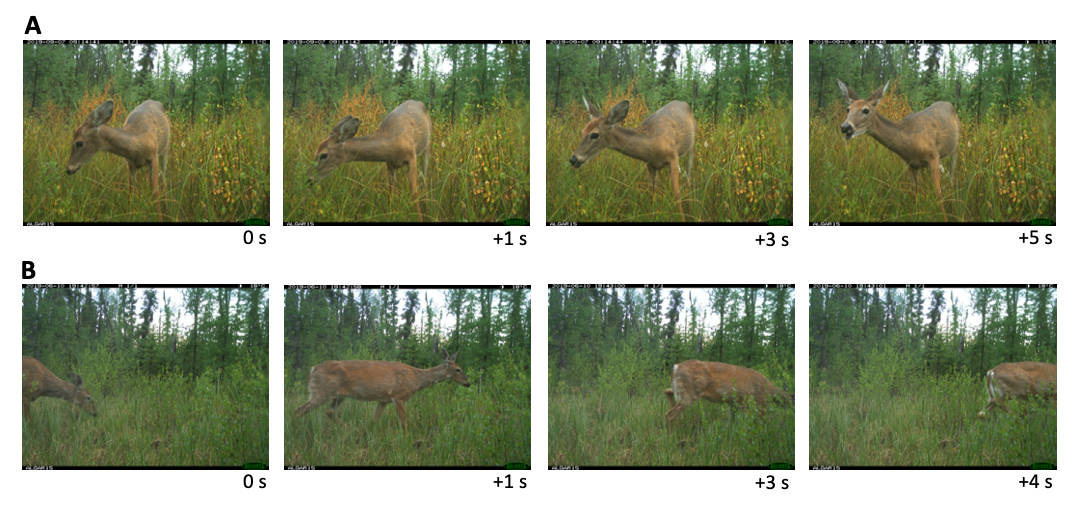
**

**Figure S1.** Excerpts from example CT image sequences contrasting between a detection event considered to reflect more secure behavior (foraging, A), and one considered to be more reflective of risk-averse behavior (travelling, B) for white-tailed deer (*Odocoileus virginianus*) in the Algar study area in northeastern Alberta, Canada. Event A consisted of a sequence of 33 images with a total duration of 4 minutes and 23 seconds and contained a juvenile, while event B had only 5 images and a duration of 10 seconds. Sequence excerpts shown here are consecutive images, with time elapsed in seconds from the first image shown in the bottom corner.

**Independence threshold for detection events**

To define an independent detection event, we determined the time threshold that best separated distinct sequences of detections of the same species. Specifically, we plotted the distribution of times between consecutive images of a species (for white-tailed deer, moose, caribou, black bear, and wolf) to identify a cut-off time within which the majority of consecutive images occurred (Fig S2). The majority of consecutive images of a species occurred within a few minutes of each other. We conservatively used an interval of 15 minutes to define the maximum amount of elapsed time between consecutive images before assigning them to independent detection events.

**
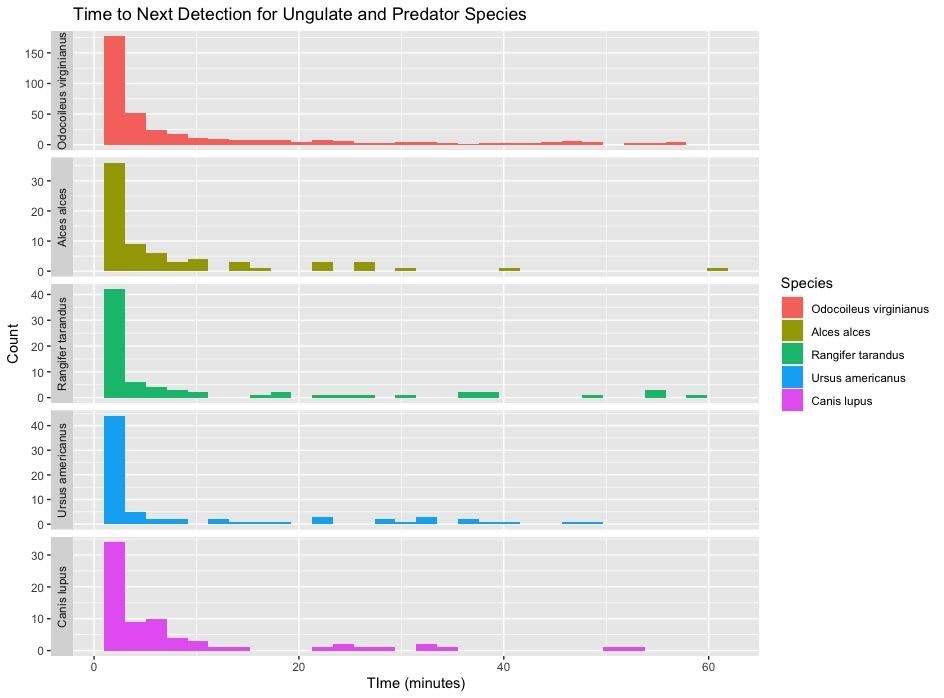
**

**Figure S2.** Frequency distribution of elapsed times to next detections for consecutive images of the same species, for white-tailed deer (*Odocoileus virginianus*), moose (*Alces alces*), caribou (*Rangifer tarandus*), black bear (*Ursus americanus*), and wolf (*Canis lupus*). The frequency of consecutive images of a species dropped and levelled out by approximately 15 minutes, indicating a natural threshold with which to delineate independent detections.

**Classifying ungulate behaviors within detection events**

In considering the response variable based on classifying observed ungulate behaviors, we sought to determine if behaviors were consistently classified as either "secure" or "travelling" across all images within an independent event (i.e. up to 15 minutes between consecutive images, as described above). In cases where they were not, we evaluated how robust classifications at the event level were to variation among images by comparing the mean and mode classification among images within an event (with "secure" classed as 1 and "travelling" as 0). We also compared a classification for which “secure” was used at the event level if there was at least one or more images classified as “secure” within the event. We plotted the frequency distribution of event classifications (for all 3 ungulate species combined, Fig. S3, and each species individually, Figs. S4-S6). Despite some variation in classifications among images within events, the overall behavioral classifications at the event level appeared to be robust to this variation.


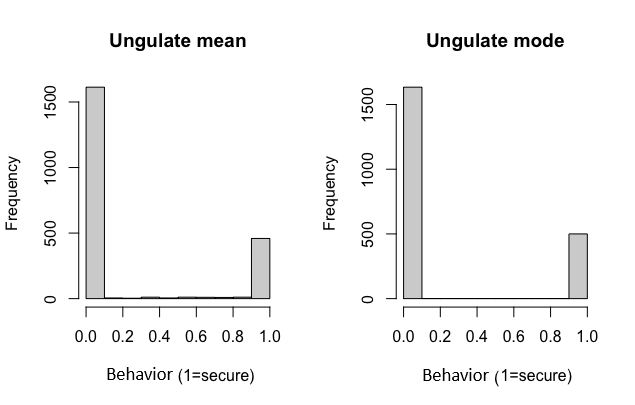


**Figure S3.** Frequency histograms of ungulate behavior classifications (grouped across caribou, moose, and white-tailed deer) among images within independent detection events. Mean is the average of images classified as secure (1) or travelling (0) behaviors within each event. Mode is the most frequent behavior classified in each event.

**
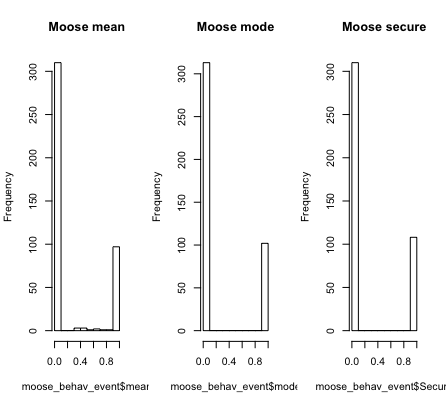
**

**Figure S4.** Frequency histograms of moose behavioral classifications across images within detection events. Mean is the average of images classified as secure (1) or travelling (0) behaviors within each event. Mode is the most frequent behavior classified in each event. Secure is the count of events classified as secure (1) or travelling (0) when secure was defined for the event if at least one image was classified as demonstrating secure behavior.

**
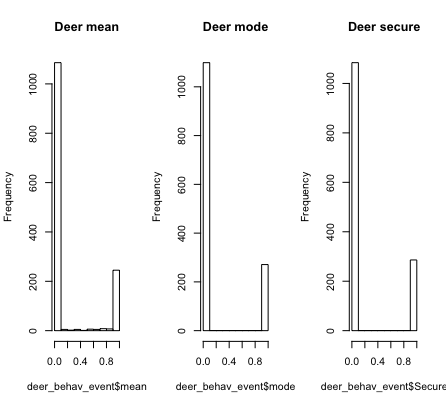
**

**Figure S5.** Frequency histograms of white-tailed deer behavioral classifications across images within detection events. Mean is the average of images classified as secure (1) or travelling (0) behaviors within each event. Mode is the most frequent behavior classified in each event. Secure is the count of events classified as secure (1) or travelling (0) when secure was defined for the event if at least one image was classified as demonstrating secure behavior.

**
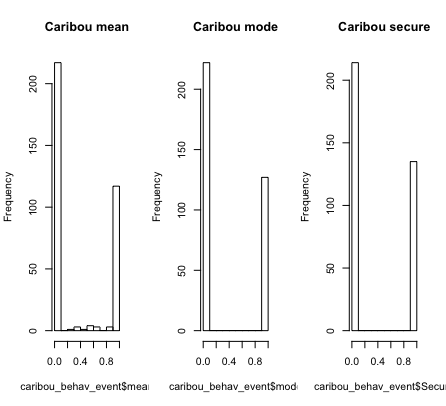
**

**Figure S6.** Frequency histograms of caribou behavioral classifications across images within detection events. Mean is the average of images classified as secure (1) or travelling (0) behaviors within each event. Mode is the most frequent behavior classified in each event. Secure is the count of events classified as secure (1) or travelling (0) when secure was defined for the event if at least one image was classified as demonstrating secure behavior.

**Correlations among ungulate behavior response variables**

We explored correlations among the three behavioral response variables for the three ungulate species (white-tailed deer, caribou, and moose). Specifically, we used a linear model to test correspondence between the number of photos in each event and the event duration (in minutes; with both variables log-transformed). We also compared the mean number of photos per event between events classified as Secure vs. Travelling.

The number of photos per event and event duration were both right-skewed (Fig. S7), with most events having few photos and being short in duration. The two variables were strongly positively correlated (R^2^ = 0.72; Fig. S8). Events classified as Secure also had significantly more photos than events classified as Traveling, across all three ungulates (β=15.45, P < 0.001; Fig. S9) and for each species (Fig. S10).


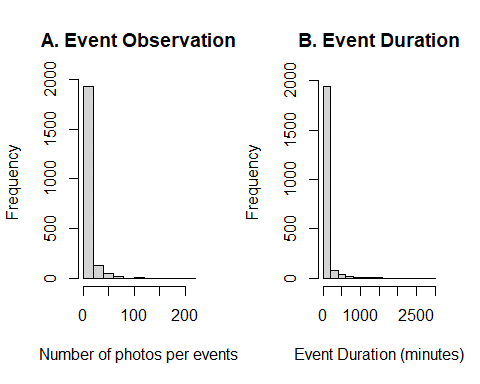


**Figure S7.** Frequency histograms of the number of photos per event (A) and the duration of events (B) across all three focal ungulate species (caribou, moose, and white-tailed deer).


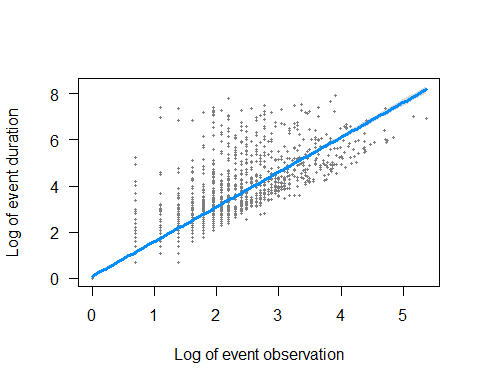


**Figure S8.** Correlation between log-scaled event observations (number of photos per detection event) and log-scaled event duration (minutes), combined across caribou, moose, and white-tailed deer.


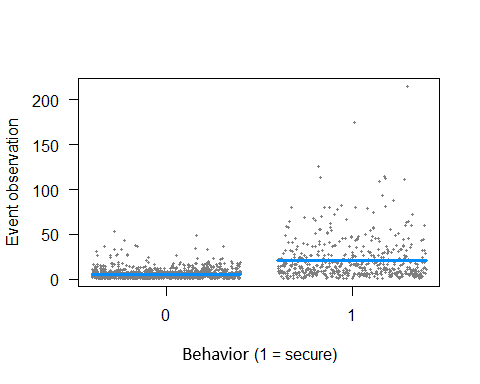


**Figure S9.** Means of event observations (photos per event) between events classified as secure (1) or travelling (0) behavior, combined across all three ungulate species.


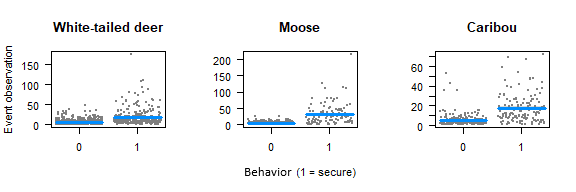


**Figure S10.** Means of event observations (photos per event) between events classified as secure (1) or travelling (0) behavior, for each species individually.

**
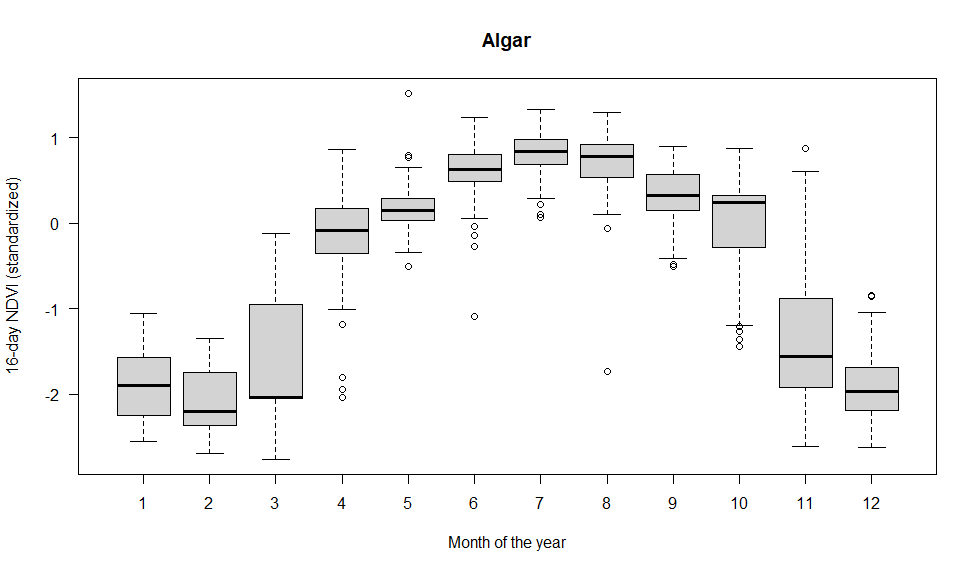
**

**Figure S11**. Boxplot of standardized 16-day NDVI values across camera trap sites within the Algar study area over the entire sampling period, summarized by month (1 = January, 12 = December).


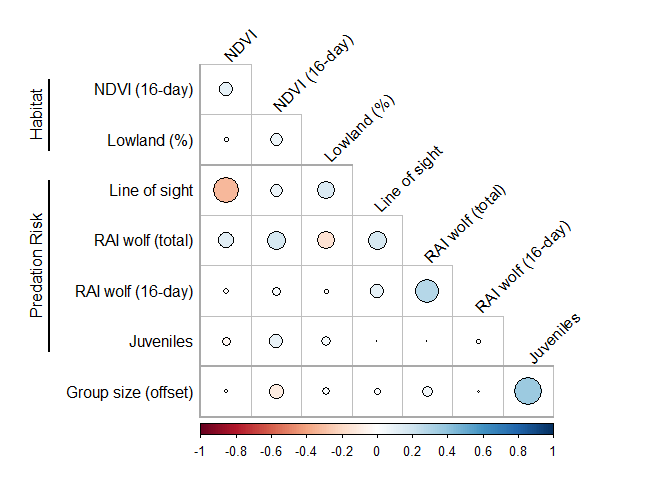


**Figure S12**. Pearson correlation coefficients between predictor variables used to model event duration (min), the probability that animals exhibited secure behavior, and number of photos per event for prey species (caribou, white-tailed deer, moose) in the Algar study area with n = 73 CTs sampled from November 2015 - November 2019. Predictor variables were categorized as reflecting variation in habitat or predation risk. Magnitude of correlations ranged from r = 0.00 - 0.37, with the greatest correlation between group size and the presence of juveniles.

**SUPPLEMENTARY RESULTS**

**Table S1.** Independent detection events by camera traps set on- vs. off of seismic lines in the Algar and Richardson study areas.

| **Species** | **Algar on-line** | **Algar off-line** | **Richardson**  **on-line** | **Richardson**  **off-line** |
| --- | --- | --- | --- | --- |
| Wolf | 502 | 8 | 15 | 19 |
| Moose | 484 | 69 | 93 | 82 |
| Caribou | 420 | 49 | 196 | 315 |
| White-tailed deer | 1501 | 343 | 6 | 5 |

**Table S2.** Posterior distributions for full period GLMM negative binomial Bayesian models assessing the influence of predation risk and habitat covariates on photos per detection event for white tailed deer, moose and caribou in the Algar study area. Specifically, we tested the influence of seismic line of sight (m), wolf detection rate (RAI) over the full study period, wolf detection rate over 16 day-periods (16-day Wolf RAI), NDVI over the full study period and also over 16 day period (16-day NDVI), percent of lowland forest (Lowland forest), and the presence of juveniles (juveniles) on each focal ungulate species. Significant effects are noted in bold based on 95% credible intervals (CI) not overlapping 0.

| Species | Parameter | Estimate | Lower 95% CI | Upper 95% CI | Rhat | Effective Sample Size |
| --- | --- | --- | --- | --- | --- | --- |
| White tailed deer | Intercept | 1.81 | 1.67 | 1.95 | 1 | 13243 |
|  | **Line of sight** | **-0.24** | **-0.39** | **-0.10** | **1** | **12519** |
|  | Wolf RAI | 0.01 | -0.09 | 0.10 | 1 | 13165 |
|  | 16-day Wolf RAI | 0.04 | -0.03 | 0.11 | 1 | 12818 |
|  | NDVI | -0.03 | -0.14 | 0.09 | 1 | 13304 |
|  | 16-day NDVI | 0.03 | -0.02 | 0.08 | 1 | 13254 |
|  | Lowland forest | -0.03 | -0.17 | 0.10 | 1 | 12892 |
|  | Juveniles | -0.16 | -0.37 | 0.04 | 1 | 13503 |
| Moose | Intercept | 2.12 | 1.87 | 2.46 | 1 | 13251 |
|  | Line of sight | -0.00 | -0.25 | 0.24 | 1 | 13416 |
|  | Wolf RAI | -0.18 | -0.39 | 0.03 | 1 | 13294 |
|  | 16-day Wolf RAI | -0.09 | -0.23 | 0.06 | 1 | 12970 |
|  | NDVI | -0.04 | -0.26 | 0.20 | 1 | 13029 |
|  | **16-day NDVI** | **0.16** | **0.05** | **0.27** | **1** | **12933** |
|  | Lowland forest | -0.04 | -0.30 | 0.22 | 1 | 12944 |
|  | **Juveniles** | **-0.40** | **-0.68** | **-0.10** | **1** | **13131** |
| Caribou | Intercept | 1.76 | 1.69 | 2.24 | 1 | 13627 |
|  | Line of sight | 0.00 | -0.21 | 0.21 | 1 | 12933 |
|  | Wolf RAI | 0.24 | -0.30 | 0.80 | 1 | 13039 |
|  | 16-day Wolf RAI | 0.05 | -0.26 | 0.39 | 1 | 13113 |
|  | NDVI | -0.02 | -0.26 | 0.23 | 1 | 12703 |
|  | 16-day NDVI | 0.06 | -0.09 | 0.20 | 1 | 13354 |
|  | Lowland forest | 0.04 | -0.23 | 0.31 | 1 | 13294 |
|  | **Juveniles** | **-0.41** | **-0.77** | **-0.03** | **1** | **13088** |


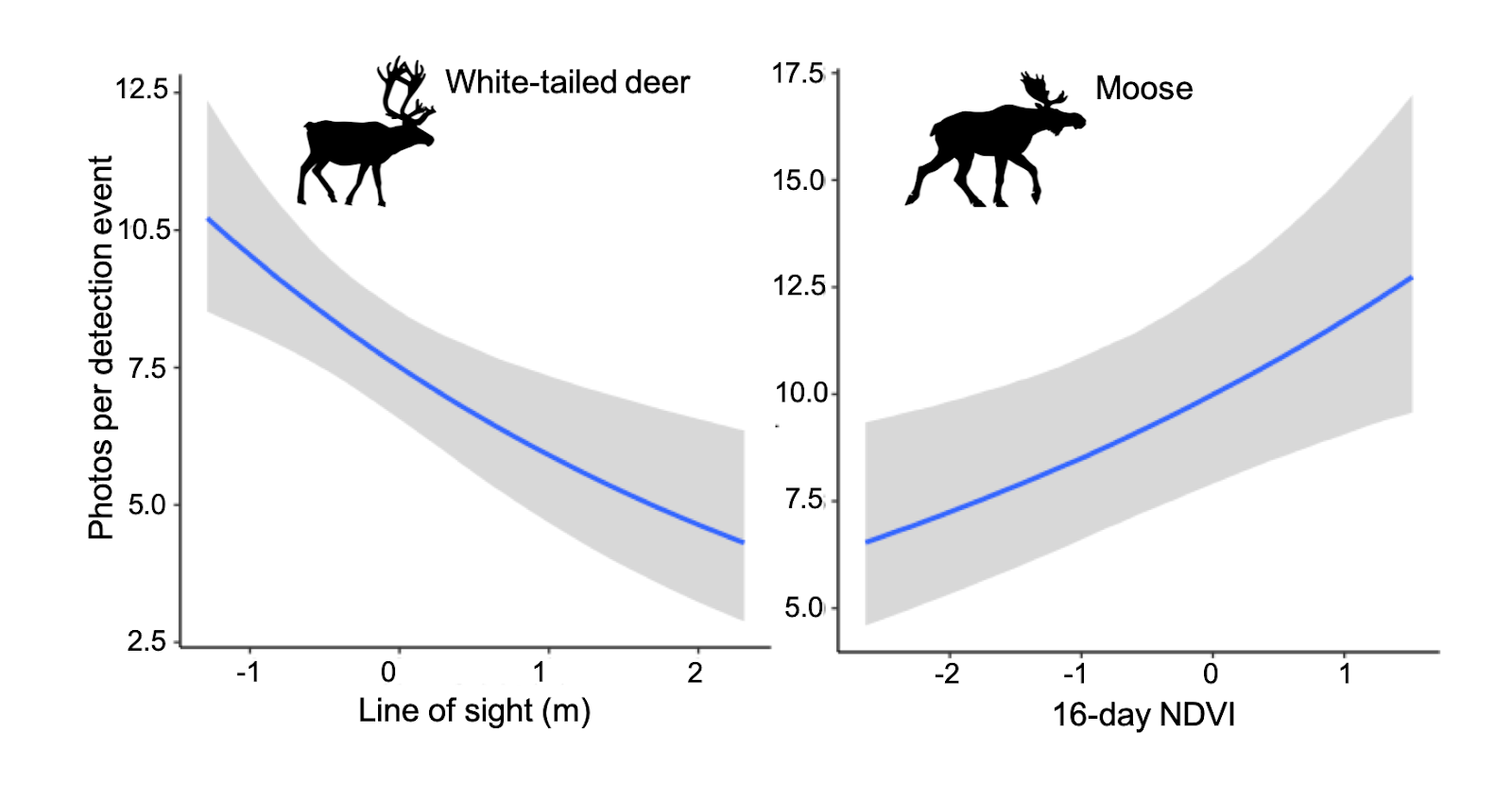


**Figure S13**. Model-predicted relationship (with 95% credible interval) between photos per detection event for white-tailed deer and line of sight (left), and for moose and 16-day NDVI (right) at CT sites within the Algar study area. Predictions are from generalized linear mixed models with other continuous predictor variables held constant at their means (wolf detection rate, percent lowland forest, NDVI, with group size as an offset variable) and juveniles present. Models were run with a negative binomial distribution with site as a random effect.

**Results using other behavior response variables:**

**Secure vs travel behavioral classifications**


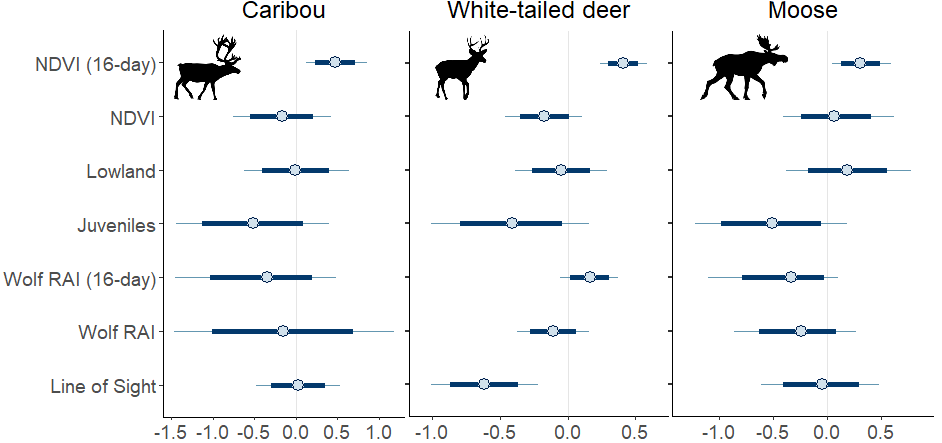


**Figure S14**. Posterior coefficient estimates for generalized linear mixed models of behavioral responses of caribou, white-tailed deer, and moose as a function of site-level predation risk (estimated by wolf relative abundance, RAI, at 16-day and full survey temporal scales, line of sight, and the presence of juveniles) and habitat quality (estimated by NDVI at 16-day and full survey temporal scales, and percent lowland forest in a 500m radius around the CT location (Lowland) on the probability that each species would exhibit secure (or travel) behavior. Secure behavior was modeled as a binary response variable (secure behavior =1, traveling behavior = 0). All predictor variables were standardized to have a mean of 0 and standard deviation of 1 (with the exception of binary presence/absence of juveniles).

**Event duration**


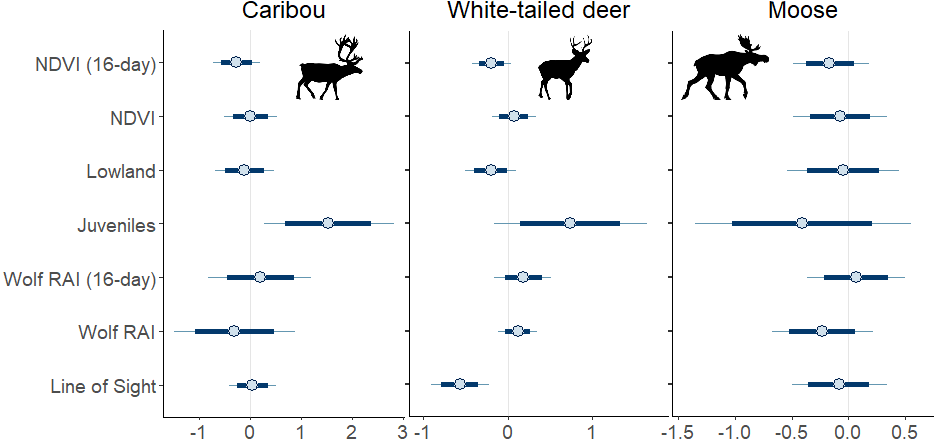


**Figure S15.** Posterior coefficient estimates for linear mixed models of behavioral event duration (min) for caribou, white-tailed deer, and moose as a function of site-level predation risk (estimated by wolf relative abundance, RAI, at 16-day and full survey temporal scales, line of sight, and the presence of juveniles) and habitat quality (estimated by NDVI at 16-day and full survey temporal scales, and percent lowland forest in a 500m radius around the CT location (Lowland). All predictor variables were standardized to have a mean of 0 and standard deviation of 1 (with the exception of binary presence/absence of juveniles).

**Model posterior predictive checks**


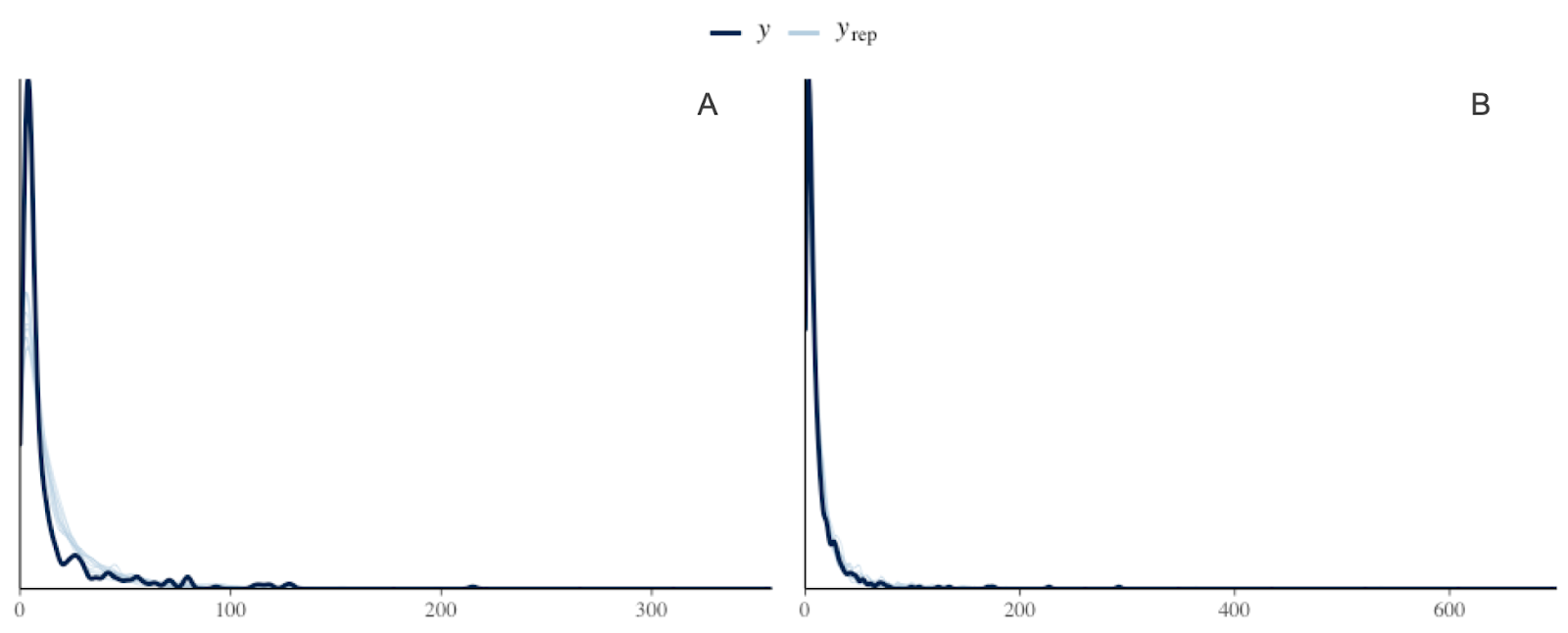


**Figure S16**. Density overlay plots for posterior predictive check tests for Bayesian GLMM models testing for differences in photos per behavioural event as the response in Algar relative to Richardson study areas. Plots are for A) moose and B) caribou. Dark blue lines (y) are the density curves for observed data and light blue lines (y_rep_) are the overlaying density curves for model simulations.


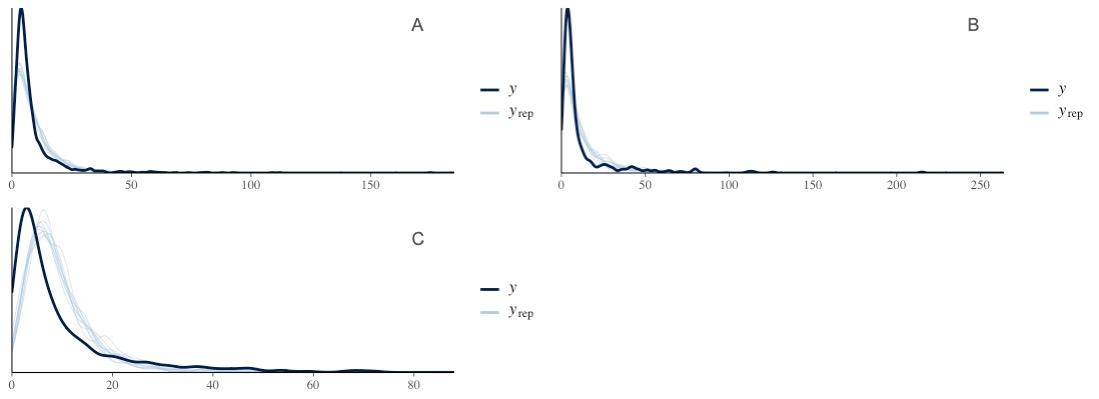


**Figure S17**. Density overlay plots for posterior predictive check tests for Bayesian GLMM with negative binomial models using photos per behavioural event as the response. Plots are for A) white-tailed deer, B) moose, and C) caribou within the Algar landscape. Dark blue lines (y) are the density curves for observed data and light blue lines (y_rep_) are the overlaying density curves for model simulations.


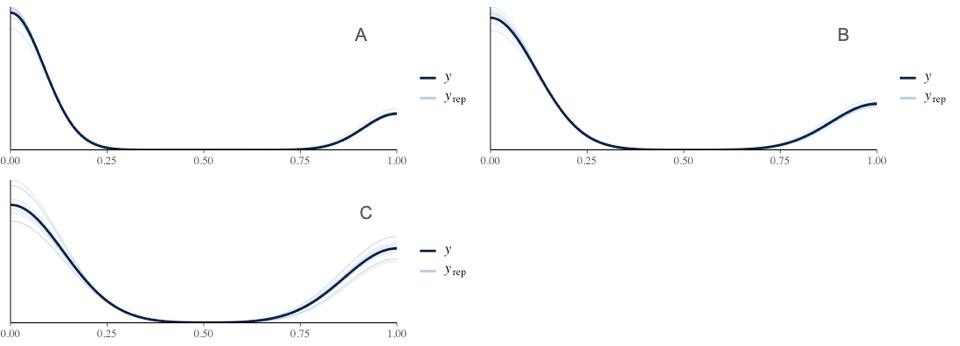


**Figure S18**.  Density overlay plots for posterior predictive check tests for Bayesian binomial models from models using secure behaviour as the response (secure/not secure). Plots are for A) white-tailed deer, B) moose, and C) caribou within the Algar landscape. Dark blue lines (y) are the density curves for observed data and light blue lines (y_rep_) are the overlaying density curves for model simulations.


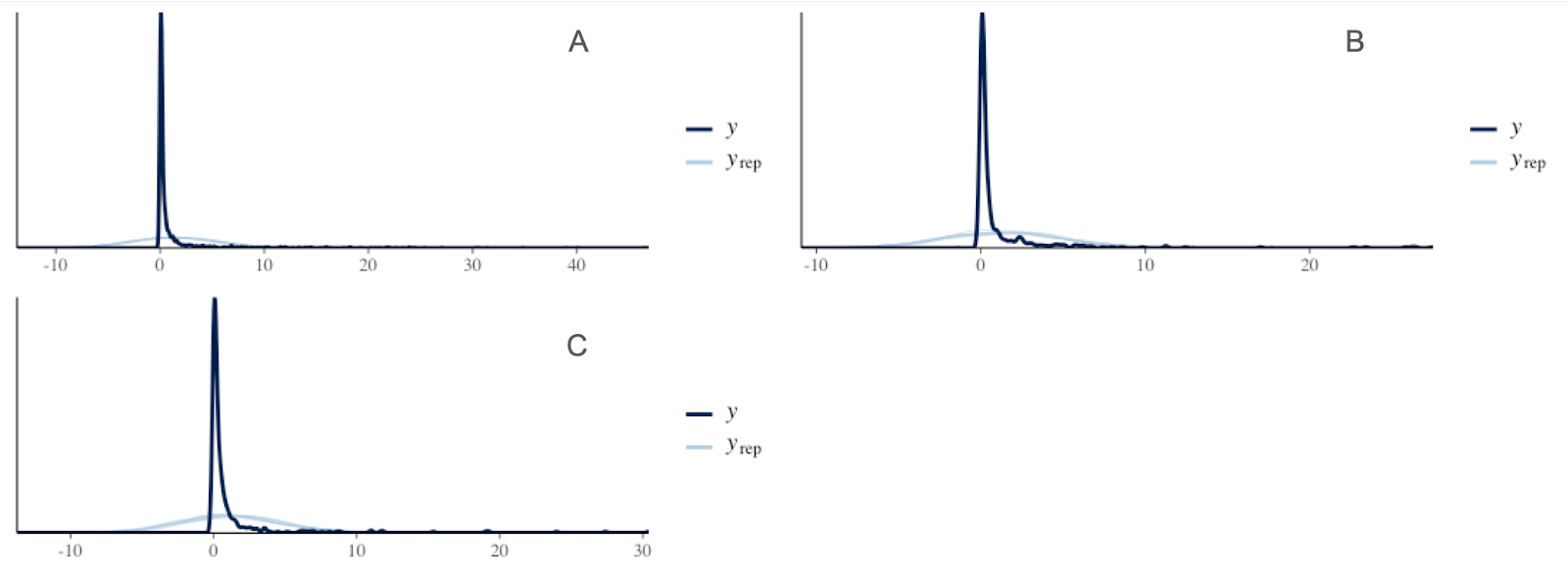


**Figure S19**. Density overlay plots for posterior predictive check tests for Bayesian LMM models using behavioural event duration as the response. Plots are for A) white-tailed deer, B) moose, and C) caribou within the Algar landscape. Dark blue lines (y) are the density curves for observed data and light blue lines (y_rep_) are the overlaying density curves for model simulations.

**Table S3**. Bayesian R-squared values for landscape-level GLMMs comparing behavioural responses (photos per event) between Algar and Richardson landscapes.

| **Species** | **R-squared** |
| --- | --- |
| Moose | 0.205 (+ 0.0354 SE) |
| Caribou | 0.353 (+ 0.0358 SE) |

**Table S4**. Bayesian R-squared value for site-level GLMMs within the Algar landscape (with different behavioural response variables)

| **Species** | **R-squared** (by response variable) | | |
| --- | --- | --- | --- |
|  | Photos per event | Secure behaviour | Event Duration |
| White-tailed deer | 0.146 (+ 0.0122 SE) | 0.112 (+ 0.016 SE) | 0.0380 (+ 0.0091 SE) |
| Moose | 0.171 (+ 0.0344 SE) | 0.151 (+ 0.0316 SE) | 0.0470 (+ 0.0187 SE) |
| Caribou | 0.366 (+ 0.053 SE) | 0.175 (+ 0.030 SE) | 0.0791 (+ 0.0226 SE) |
